# Prevalence and factors associated with common mental disorders in young people living with HIV in sub‐Saharan Africa: a systematic review

**DOI:** 10.1002/jia2.25705

**Published:** 2021-06-24

**Authors:** Ezra K Too, Amina Abubakar, Carophine Nasambu, Hans M Koot, Pim Cuijpers, Charles RJC Newton, Moses K Nyongesa

**Affiliations:** ^1^ KEMRI‐Wellcome Trust Research Programme Centre for Geographic Medicine Research (Coast) Kilifi Kenya; ^2^ Department of Public Health Pwani University Kilifi Kenya; ^3^ Department of Psychiatry University of Oxford Oxford UK; ^4^ Institute for Human Development Aga Khan University Nairobi Kenya; ^5^ Department of Clinical Neuro‐ and Developmental Psychology Amsterdam Public Health Research Institute Vrije Universiteit Amsterdam Amsterdam The Netherlands

**Keywords:** young people, HIV infections, depression, anxiety, correlates, sub‐Saharan Africa

## Abstract

**Introduction:**

Common mental disorders (CMDs) particularly depression and anxiety, are highly comorbid with HIV also in young people living with HIV (YLWH). In sub‐Saharan Africa (SSA) where most YLWH reside, there are limited summary data on CMDs among these youths, yet there are previous systematic reviews summarizing data on CMDs among adults living with HIV. We conducted a systematic literature review on the prevalence and correlates of CMDs among YLWH, aged 10 to 24 years, from SSA.

**Methods:**

We searched African Index Medicus, African Journals Online and five other electronic databases (from database inception up to 31 December 2020) for relevant studies published in English. The key search terms applied were as follows: “Depression OR Anxiety”, “Young people”, “HIV infections” and “sub‐Saharan Africa”.

**Results and discussion:**

Out of 3989 articles, 31 studies were included in the review. The prevalence of CMDs in YLWH widely varied ranging between 16.0% and 40.8% for major depression, 4.4% and 52.6% for depressive symptoms and 2.2% and 25.0% for anxiety symptoms. Anxiety disorder was estimated at 45.6%. Four of the five included studies with a comparison group of HIV‐negative young people reported significantly higher prevalence estimates of depressive disorders among YLWH. Several sociodemographic, psychosocial and HIV‐related correlates of CMDs were reported but most lacked consensus across studies. Nevertheless, female sex, older age, fewer schooling years, HIV‐positive status, bullying, sexual abuse, HIV‐related stigma, social support and poor antiretroviral therapy adherence were frequently reported (in ≥2 studies) as significant correlates of depressive symptoms among YLWH. Higher social support was the only frequent significant correlate of anxiety symptoms.

**Conclusions:**

The burden of CMDs among YLWH from SSA is substantial and appears to be significantly higher when compared with HIV‐negative peers, particularly for depressive disorders. However, more comparative research is needed. Importantly, screening for CMDs at the youth HIV‐clinics should be prioritized especially for YLWH at high risk of CMDs, to facilitate early management or referral for treatment. Furthermore, youth‐friendly psychological interventions addressing CMDs in YLWH should urgently be piloted in SSA, incorporating contextual components that may directly or indirectly reduce symptoms of CMDs among YLWH, such as social support.

## 1 Introduction

Globally, there are over 1.8 billion young people aged 10 to 24 years, the majority residing in low‐ and middle‐income countries [1]. The term “young people” generally combines the overlapping terms of “adolescents,” that is individuals in the 10‐ to 19‐year age group and “youths,” that is the 15‐ to 24‐year age group [2] and will be used to refer to both age groups in this work. Young people represent a significant proportion of people living with HIV. As of 2018, 1.6 million young people aged 10 to 19 years were living with HIV globally [3], whereas 3.9 million young people aged 15 to 24 years lived with HIV worldwide by 2014 [4]. Most of these young people were infected through vertical transmission and live in sub‐Saharan Africa (SSA) [5, 6].

Common mental disorders (CMDs), herein referring to depressive and anxiety disorders or their symptoms, are very frequent in people living with HIV [7, 8, 9] and the risk is two to three times higher than the general population [10]. Among young people living with HIV (YLWH), global reviews [5, 11, 12] report the prevalence of comorbid CMDs as high as 44.0% for depressive disorders and 48.2% for anxiety disorders. In recent reviews on the burden of psychiatric disorders among YLWH aged 10 to 19 years in SSA [13, 14], depressive and anxiety symptoms ranged from 14% to 53% and 15% to 25% respectively. The burden of CMDs may also be higher in YLWH than their peers without HIV [15] or even other vulnerable groups of young people, such as those in juvenile detention [16]. The higher risk of CMDs in people living with HIV, including the youth, may be caused by side effects of antiretroviral therapy (ART) [17, 18], persistent HIV‐related stigma in the community [19, 20], the direct and indirect neurologic effects of HIV on the brain [21] and the fear of premature death [22]. There are detrimental consequences when CMDs co‐occur with HIV including worsened prognosis of HIV infection [23], increased risk of suicidality [24], non‐adherence to ART [25], poor quality of life [26] and alteration of economic productivity of people living with HIV [23].

Previous global reviews [5, 11, 12] and one recent review from SSA [13] have reported on factors associated with CMDs among YLWH, but there is little or no consensus across individual studies included in these reviews. Notably, most of the studies included in the global reviews have been conducted in Western countries (Europe and North America). Only four out of the 14 studies included in the review from SSA reported a few correlates of CMDs. Nevertheless, female sex [16, 27], older age [28, 29, 30], poor adherence to ART [31, 32], stressful life events [33, 34], parental or caregiver mental health status [15, 27, 35], maternal HIV‐positive status [15, 29], low cluster of differentiation‐4 (CD4) cell count [27, 36] and history of AIDS‐defining illness [36, 37] appear important correlates of CMDs in YLWH as they are reported by more than one study in the aforementioned reviews. Extrapolating findings from especially reviews of global nature to inform interventions seeking to address the mental health of YLWH from settings such as SSA may be problematic because of contextual differences (especially where very few of the studies included are from the setting targeted for intervention). With the increasing research effort towards an understanding of the mental health of people living with HIV from SSA [38], including the youth [39, 40, 41], there is a need for a greater understanding of context‐specific factors associated with CMDs among people living with HIV from this setting.

The increasing mental health issues among YLWH is an emerging public health concern with the potential of burdening the already busy healthcare systems and the scarce human resources in mental healthcare in resource‐limited settings like those of SSA [7, 23]. Hitherto, no study has extensively summarized data on the burden and contextual determinants of CMDs among YLWH from SSA, a region where most of these HIV‐positive young people reside. While there have been several global reviews trying to understand the burden of mental health problems among YLWH [5, 11, 12], most of the studies included have been conducted outside SSA, limiting the generalizability of their findings to the African context. Past systematic reviews on the burden of CMDs among people living with HIV from SSA [9, 38, 42] have only considered studies recruiting adults living with HIV sidelining YLWH who currently represent a considerable percentage of people living with HIV. The recent systematic reviews involving YLWH from SSA [13, 14] are limited to young people aged 10 to 19 years only and broadly focus on many psychiatric disorders. For the current systematic review, the overall objective was to summarize the available evidence on the prevalence and factors associated with CMDs among YLWH aged 10 to 24 years from SSA. The specific objectives of this review were:

1. To systematically summarize the existing literature on the prevalence of CMDs, specifically depressive and anxiety disorders, or their symptoms, among YLWH aged 10 to 24 years from SSA alongside information on measurement tools used and their contextual reliability and/or validity.
2. To systematically identify the factors associated with CMDs, specifically depressive and anxiety disorders or their symptoms, among YLWH aged 10 to 24 years from SSA.

The added value of this review is two‐fold. First, by extending the review age range, we include young people regarded as adults (societally and/or by law) who are expected to take care of themselves, most often outside the family context, with implications for an arrangement of mental health support. Second, although the recent reviews from SSA give important information on the prevalence and range of mental disorders in YLWH, the review by Olashore *et al*. [14] only addresses the association of these disorders with ART adherence. In both reviews [13, 14], their approach limits the possibility to understand which contextual factors importantly relate to CMDs, the most frequent of the psychiatric disorders. This review addresses this gap.

## 2 Methods

### 2.1 Search strategy

The Preferred Reporting Items for Systematic Review and Meta‐analysis (PRISMA) guidelines [43] informed the design and reporting of this systematic review. The study protocol was registered with the International Prospective Register of Systematic Reviews (PROSPERO) under registration number CRD42020160806. Structured electronic searches were initially conducted in African Index Medicus, African Journals Online, Google Scholar, PsycArticles, PsycInfo, PubMed and Web of Science Core collection databases between 3 and 24 December 2019. The search was later updated in January 2021 to capture publications up to 31^st^ December 2020. Where applicable, databases were searched from the time of their inception. The key search terms included “Depression/Anxiety”, “Young people”, “HIV infections” and “sub‐Saharan Africa” which were combined by the Boolean operator “AND”. Synonyms for each of the key search terms were combined using the “OR” Boolean operator. Where applicable, Medical Subject Headings (MeSH) terms were used. (Additional file S1) provides the search string used in the PubMed database, which was modified to meet the specifications for other databases.

The search was restricted to studies published in the English language, where a database could allow this filter. All the retrieved references were exported and managed in EndNote version 7. An additional search for relevant articles was conducted by scanning the reference lists of included articles and any relevant systematic reviews captured in the initial search.

### 2.2 Screening of articles by inclusion and exclusion criteria

For potential inclusion, all the articles returned from the database searches were screened in two steps: i) based on title and abstract and ii) by full text. The first author (ET) screened the articles for eligibility. To reduce bias that may arise from human error, two other reviewers (CN and MKN) independently repeated 10% of the study screening at every stage through a systematic random selection of articles divided into two halves, each for the independent reviewers. Rates of agreement between each of the independent reviewers and the first reviewer were consistently high, and minor disagreements were settled through consensus. To be included for review, studies had to fulfil a pre‐determined inclusion and exclusion criteria as shown in Table 1.

**Table 1:** Study selection criteria

Inclusion criteria	Exclusion criteria
Population	
‐ Studies with HIV‐positive young people from SSA. ‐ Studies with participants aged 10 to 24 years or with mean/median age within this age bracket.	‐ Studies involving HIV‐positive young people outside SSA. ‐ Studies involving HIV‐negative young people or with HIV‐positive participants outside the 10 to 24 age range. ‐ Studies with unspecified age range, mean or median age of participants. ‐ Studies with very specific subpopulations of young people e.g. pregnant women, out‐of‐school adolescents et cetera.
Outcome	
‐ Studies on depression or depressive symptoms and associated factors. ‐ Studies on anxiety disorder or anxiety symptoms and associated factors.	‐ Studies on mental disorders other than depression or anxiety, or their symptoms. ‐ Studies using measurement scales evaluating both anxiety and depression without providing separate data. ‐ Studies where reported measurement scales do not evaluate depression or anxiety, or their symptoms.
Study designs	
‐ Cross‐sectional studies ‐ Case‐control studies ‐ Cohort studies	‐ Review articles e.g. narrative, systematic, scoping ‐ Intervention studies ‐ Case studies/reviews ‐ Commentaries or editorials ‐ Conference proceedings, symposia abstracts, or workshop publications ‐ Qualitative studies ‐ Books or book sections ‐ Theses and dissertations

Studies with a comparison group of HIV‐negative young people and providing disaggregated mental health data by HIV infection status were included. Studies duplicating similar project data to an already included main and more comprehensive article were excluded. SSA, sub‐Saharan Africa.

### 2.3 Data extraction

ET and MKN independently extracted the following data from included studies using a standardized Microsoft Excel data abstraction form: (i) Article details – name of the first author, title and publication year; (ii) Study details – country of origin, study design, study setting, data collection period and response rate (where reported) and the reported study limitations; (iii) Study participant characteristics – HIV‐related data (mode of acquisition, time since diagnosis, ART regimen and duration on ART treatment), sample size, sampling method, source of the sample, age (mean, median, or range) and sex (proportion of male vs. female); (iv) Outcome and measures – prevalence of depression and anxiety, or their symptoms, measurement tool used, cut off score applied (for screening tools), information on local tool validation, factors associated with depression and anxiety or their symptoms (alongside the reported measure of effect and precision estimate).

### 2.4 Quality assessment

ET and MKN independently assessed the risk of bias of each included study using the Newcastle–Ottawa Scale [44]. These authors then held a meeting to resolve any disagreements in quality rating. Using this tool, studies were assessed based on three domains: the selection of participants, comparability of study groups and the ascertainment of exposure (for case–control studies) or outcome of interest (for cohort and cross‐sectional studies). A star‐grading system was used, with each domain item receiving one or two stars if appropriate methods were reported. A maximum of nine stars was awarded for cohort and case–control studies, and a maximum of 10 stars for cross‐sectional studies. Studies were classified as unsatisfactory, satisfactory, good and very good if they had a total of 0 to 4, 5 to 6, 7 to 8 and 9 to 10 stars respectively.

### 2.5 Data analysis

Because of the heterogeneous nature of measurement tools used across included studies, data were narratively summarized. Data on the prevalence of CMDs among YLWH were summarized by each homogeneous measurement tool used, whereas data on correlates of CMDs were summarized by the investigated outcome (depression or anxiety). We manually calculated the odds ratio using the reported proportions from individual studies comparing the prevalence of CMDs between YLWH and their HIV‐negative peers. In this review, only factors significantly associated with either depression or anxiety, or their symptoms at *p* < 0.05 in the multivariable analysis were considered and extracted as correlates. Basic descriptive statistics (frequencies with percentages) were used to summarize data on the region of SSA where each of the included studies was conducted.

## 3 Results and Discussion

**Figure 1:**
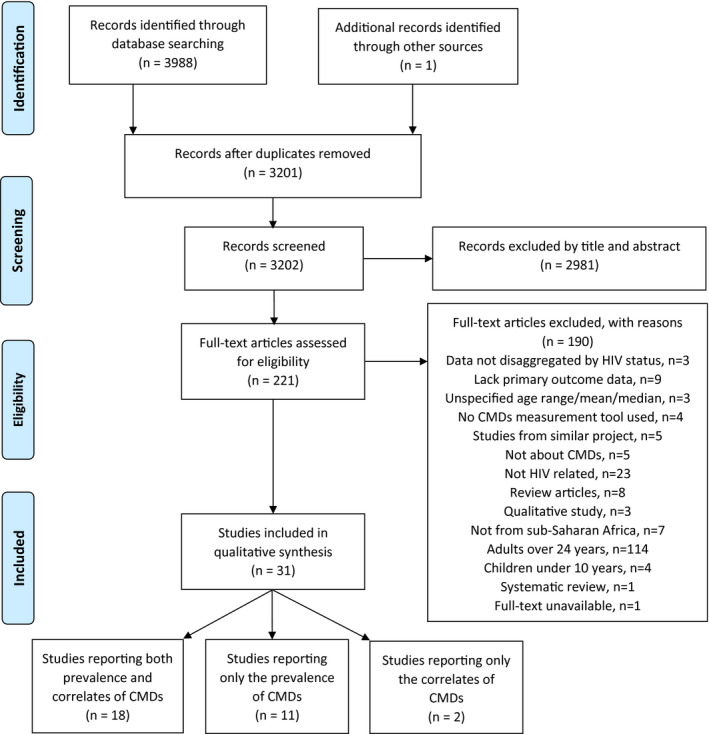
PRISMA flowchart for the systematic review process. CMDs, common mental disorders.

The electronic search yielded 3988 hits from the different databases (African Index Medicus, n = 11, African Journals Online, n = 147, Google Scholar, n = 2137, PsycArticles, n = 8, PsycInfo, n = 865, PubMed, n = 302 and Web of Science, n = 518). A scan of the reference lists of included articles and relevant systematic reviews captured by the search yielded one additional article. After removing duplicates and screening articles based on the eligibility criteria, we included 31 studies in the review. Figure 1 shows the PRISMA flowchart for the systematic review process. CMDs, common mental disorders.

### 3.1 Characteristics of included studies

(Additional file S2) presents in detail the characteristics of the 31 included studies. The reviewed studies were mostly conducted in Eastern (n = 13; 41.9%) or Southern (n = 13; 41.9%) African countries except four studies [45, 46, 47, 48] that were conducted in a Western African country (12.9%) and one study (3.2%) from Central Africa [49]. The included studies enrolled a total of 9935 YLWH (individual study sample size ranging from 58 to 1088). Additionally, the studies with a comparison group enrolled 1000 HIV‐negative young people (individual study sample size ranging from 44 to 600).

Many of the studies (n = 29) were cross‐sectional in design and published after 2010, except two studies [47, 50]. A review of the literature on depression among HIV‐positive adults from SSA [38] also observed that most of the studies included were conducted after 2010. It is, therefore, encouraging to note that from the beginning of the last decade, there is an upsurge of research work towards an understanding of the mental health of people living with HIV in Africa with the potential to inform clinical practice and policy.

Many of the studies were conducted in urban settings of SSA (n = 20). Nearly all studies (n = 29) recruited YLWH from HIV‐specialized clinics except one study [51] that recruited from the community. In one study [52], this information could not be retrieved because the study was only available in abstract form (see study quality section for details). YLWH were exclusively on ART in most studies (n = 17). Eight studies [46, 53, 54, 55, 56, 57, 58, 59] recruited ART experienced (majority) and ART naïve participants, whereas six studies [45, 48, 60, 61, 62, 63] did not provide information on participant ART‐use status. In eight studies [52, 55, 59, 61, 64, 65, 66, 67] YLWH were exclusively perinatally HIV‐infected. Five studies [45, 49, 58, 68, 69] had a mixture of perinatally and behaviourally HIV‐acquired youths; the rest (18 studies) did not provide information on the mode of HIV infection. Outside Africa, certain characteristics of YLWH like mode of HIV infection (behavioural vs. perinatal) [8, 70] and ART‐use status (ART naïve vs. ART experienced) [38] may influence their mental health experiences. In this review, none of the studies recruiting a mix of participants provided disaggregated mental health data by any of these characteristics. An in‐depth investigation of this nature in the African context will require researchers working with a mixed sample of YLWH to collect and profile disaggregated mental health data by, for example mode of HIV infection or ART‐use status. Additional data on CMDs among YLWH residing in rural settings of SSA are also needed, so far, research focus has been biased towards urban settings.

### 3.2 Measurement tools for CMDs, their reliability and validity among YLWH from SSA

CMDs in YLWH from SSA were assessed using both diagnostic tools and symptom screeners. Diagnostic tools used in this study included the Mini‐International Neuropsychiatric Interview for children and adolescents (MINI‐KID) [71] used in three studies [45, 46, 64] to diagnose major depression and the tenth revision of the International Classification of Diseases (ICD‐10) symptom checklist [72] used by Musisi and Kinyanda [50] to diagnose major depression and anxiety disorder. The other studies used different types of CMD symptom screeners. Kinyanda *et al*. [55] used the 5^th^ edition of the Diagnostic and Statistical Manual (DSM) of mental disorders referenced Child and Adolescent Symptom Inventory‐5 (CASI‐5) [73] and the fourth revision of the Youth Inventory (YI‐4R) [74] to assess symptoms of major depression, any anxiety disorder, generalized anxiety disorder, social and separation anxiety disorders. Buckley *et al*. [65] used the DSM (4^th^ edition) referenced 84‐item Patient Health Questionnaire for Adolescents (PHQ‐A) [75] to assess symptoms of major depression and anxiety disorder (specifically panic disorder). Various screening tools based on different cut‐off scores were also used to measure depressive (Table 2) or anxiety symptoms (Table 3). These screening tools included the 9‐item patient health questionnaire [76] used in six studies [49, 53, 57, 60, 77, 78], the centre for epidemiologic studies depression scale [79] used in four studies [51, 54, 56, 62], the child depression inventory [80] used in four studies [58, 59, 81, 82], Beck’s depression inventory [83] used in three studies [48, 61, 84], the revised children’s depression rating scale [85] used by Kim *et al*. [28], the hospital anxiety and depression scale [86] used by Sale & Gadanya [47], Reynold’s adolescent depression Scale [87] used by Paul *et al*. [63], National Institute of Health toolbox – Sadness module [88] used by Molinaro *et al*. [52], the Beck’s youth inventory [89] used in two studies [66, 67] and the revised children’s manifest anxiety scale [90] used in two studies [58, 59]. Most studies did not report information on the reliability and/or validity of these measurement tools among YLWH. In some studies, where this information was not provided, authors pointed out that the tool they used was previously validated in the study country or provided a reference to the tool validation process (see Tables 2 and 3). Where reported, information on tool reliability and/or validity was mostly limited to Cronbach’s alpha, a measure of internal consistency of a scale, and values were above the acceptable threshold of 0.7.

**Table 2:** Prevalence estimates for major depression and depressive symptoms among YLWH from SSA according to the measurement tool used

Author, year	Country	Outcome of interest	Sample size (n)	Assessment tool used	Cut‐off score	Information on local tool validation	Prevalence estimates
The mini‐international neuropsychiatric interview for children and adolescents (MINI‐KID)							
Adeyemo *et al*., 2020 [45]	Nigeria	Major Depression	201	MINI‐KID	NA	–	16.9%
Ashaba *et al*., 2018 [64]	Uganda	Major Depression	224	MINI‐KID	NA	–	16.0%
Ashaba *et al*. [46]	Nigeria	Major Depression	75 HIV+ 75 HIV−	MINI‐KID	NA	–	20.0% among HIV+ 6.7% among HIV− *p* = 0.01
International classification of diseases, tenth edition (ICD‐10) symptom checklist							
Musisi & Kinyanda, 2009 [50]	Uganda	Major Depression	82	ICD‐10	NA	**–**	40.8%
The youth inventory fourth revision (YI‐4R) and the child and adolescent symptom inventory‐5 (CASI‐5)							
Kinyanda *et al*., 2019 [55]	Uganda	Symptoms of Major Depression	479	YI‐4R CASI‐5	NR	Cronbach alpha of 0.88 and test–retest reliability of 0.2, *p* < 0.01 Cronbach alpha of 0.77 and test–retest reliability of 0.17, *p* < 0.01	5.2%
The patient health questionnaire for adolescents (PHQ‐A)							
Buckley *et al*., 2020 [65]	South Africa	Symptoms of Major Depression	81 HIV+ 81 HIV−	PHQ‐A	NR	NR	6.2% among HIV+ 7.4% among HIV− *p* = 0.99
The 9‐item patient health questionnaire (PHQ‐9)							
Dow *et al*., 2016 [53]	Tanzania	Depressive symptoms	182	PHQ‐9	≥10	NR	12.1%
Dyer *et al*., 2020 [60]	Kenya	Depressive symptoms	479	PHQ‐9	≥5	NR	10.0%
Ekat *et al*., 2020 [49]	DRC	Depressive symptoms	135	PHQ‐9	≥9	NR	38.5%
Gaitho *et al*., 2018 [77]	Kenya	Depressive symptoms	270	PHQ‐9	≥1	NR	52.6%
Haas *et al*., 2020 [78]	South Africa	Depressive symptoms	1088	PHQ‐9	≥10	NR	4.4%
Ramos *et al*., 2018 [57]	Tanzania	Depressive symptoms	280	PHQ‐9	≥10	NR	20.4%
The centre for epidemiologic studies depression scale (CES‐D)							
Fawzi *et al*., 2016 [51]	Rwanda	Depressive symptoms	193	CES‐D	≥30	Not provided in this study. However, the authors note that they used a CES‐D previously validated in Rwanda	26.0%
Filiatreau *et al*., 2020 [62]	South Africa	Depressive symptoms	334	CES‐D	≥16	Cronbach alpha of 0.76	27.5%
Kemigisha *et al*., 2019 [54]	Uganda	Depressive symptoms	336	CES‐D	≥15	Cronbach alpha of 0.85	45.8%
Okawa *et al*., 2018 [56]	Zambia	Depressive symptoms	190	CES‐D (10‐item)	≥10	Cronbach alpha of 0.74	25.3%
The child depression inventory							
Lwidiko *et al*., 2018 [82]	Tanzania	Depressive symptoms	300 HIV+ 600 HIV−	CDI‐II	≥12	Cronbach alpha of 0.7	27.0% among HIV+ 5.8% among HIV− *p* < 0.001
Cavazos‐Rehg *et al*., 2020 [81]	Uganda	Depressive symptoms	675	CDI‐S (Short form)	≥3	Authors claim to have used culturally adapted tools	50.3%
West *et al*., 2018 [58]	South Africa	Depressive symptoms	278	CDI‐S (Short form)	≥7	Not provided in this study, but authors note that the tool was previously validated in South Africa	7.6%
Woollett *et al*., 2017 [59]	South Africa	Depressive symptoms	343	CDI‐S (Short form)	≥10	Not provided in this study, but authors say they used measures previously validated among youth in South Africa (Cronbach alpha >0.70)	14.0%
Beck’s depression inventory‐II (BDI‐II)							
Abebe *et al*., 2019 [84]	Ethiopia	Depressive symptoms	507	BDI‐II	≥21	NR	35.5%
Earnshaw *et al*., 2018 [61]	South Africa	Depressive symptoms	250	BDI‐II	≥20	Cronbach alpha of 0.9	33.8%
Yarhere & Jaja, 2020 [48]	Nigeria	Depressive symptoms	58	BDI‐II	≥11	NR	44.8%
Beck’s youth inventory‐II (BYI‐II)							
Hoare *et al*., 2019 [66]	South Africa	Depressive symptoms	204 HIV+ 44 HIV−	BYI‐II (Depression inventory)	NR	NR	6.4% among HIV+ 2.3% among HIV− *p* < 0.01
Kikuchi *et al*., 2017 [67]	Rwanda	Depressive symptoms	475	BYI‐II (Depression inventory)	>55	Cronbach alpha of 0.84	22.1%
The revised children’s depression rating scale (CDRS‐R)							
Kim *et al*., 2015 [28]	Malawi	Depressive symptoms	562	CDRS‐R	≥55	Not provided in this study. Authors provide a reference for information on tool validation	18.9%
The hospital anxiety and depression scale (HADS)							
Sale & Gadanya, 2008 [47]	Nigeria	Depressive symptoms	162	HADS (Depression scale)	≥8	NR	39.5%
Reynolds adolescent depression scale‐second edition (RADS‐2)							
Paul *et al*., 2015 [63]	Zambia	Depressive symptoms	100	RADS‐2	≥76	NR	19.0%
NIH toolbox sadness module							
Molinaro *et al*., 2019^†^ [52]	Zambia	Depressive symptoms	200 HIV+ 200 HIV−	NIH	≥60	NE	24.0% among HIV+ 13.0% among HIV− *p* = 0.03

^†^ This work was only available as a published abstract from an annual meeting with prevalence data within the abstract. DRC, Democratic Republic of Congo; NA, Not Applicable; NE, Not Extracted; NR, Not Reported.

**Table 3:** Prevalence estimates for anxiety disorder or its symptoms among YLWH from SSA according to the measurement tool used

Author, year	Country	Outcome of interest	Sample size	Assessment tool used	Cut‐off score	Information on local tool validation	Prevalence estimates
International classification of diseases, tenth edition (ICD‐10) symptom checklist							
Musisi & Kinyanda, 2009 [50]	Uganda	Anxiety disorder	82	ICD‐10	NA	–	45.6%
The patient health questionnaire for adolescents (PHQ‐A)							
Buckley *et al*., 2020 [65]	South Africa	Anxiety disorder symptoms (Panic disorder)	81 HIV+ 81 HIV−	PHQ‐A	NR	NR	3.7% among HIV+ 2.5% among HIV− *p* = 0.99
The revised children’s manifest anxiety scale (RCMAS)							
West *et al*., 2018 [58]	South Africa	Anxiety symptoms	278	14‐item RCMAS	≥10	Not provided in this study, but authors say tools were previously validated in South Africa	6.7%
Woollet *et al*., 2017 [59]	South Africa	Anxiety symptoms	343	28‐item RCMAS	NR	Not provided in this study, but authors say they used measures previously used with youth in South Africa (Cronbach alpha >0.75)	25.0%
The youth inventory fouth revision (YI‐4R) and the child and adolescent symptom inventory‐5 (CASI‐5)							
Kinyanda *et al*., 2019 [55]	Uganda	Symptoms of: ‐Any anxiety disorder ‐GAD ‐SAD ‐SEAD	479	YI‐4R CASI‐5	NR	Cronbach alpha of 0.88 and test–retest reliability of 0.2, *p* < 0.01 Cronbach alpha of 0.77 and test–retest reliability of 0.17, *p* < 0.01	14.7% for Any anxiety disorder 7.2% for GAD 7.0% for SAD 5.4% for SEAD
The 7‐item generalized anxiety disorder scale (GAD‐7)							
Haas *et al*., 2020 [78]	South Africa	Anxiety symptoms	1088	GAD‐7	≥10	NR	2.2%
Beck’s youth inventory‐II (BYI‐II)							
Hoare *et al*., 2019 [66]	South Africa	Anxiety symptoms	204 HIV+ 44 HIV−	BYI‐II (Anxiety inventory)	NR	NR	11.8% among HIV+ 9.1% among HIV− *p* = 0.61

GAD, Generalized Anxiety Disorder; NA, not applicable; NR, not reported; SAD, Social Anxiety Disorder; SEAD, Separation Anxiety Disorder.

For any meaningful epidemiological data that can inform appropriate interventions, there is a need for future studies involving YLWH in SSA to measure CMDs using culturally appropriate and locally validated tools. Where feasible, validated mental health diagnostic measures should be administered concurrently to check the diagnostic accuracy of these mental health screening tools.

### 3.3 Prevalence of CMDs among YLWH from SSA

Twenty‐nine studies reported the prevalence of either major depression or depressive symptoms [45, 46, 47, 48, 49, 50, 51, 52, 53, 54, 55, 56, 57, 58, 59, 60, 61, 62, 63, 64, 65, 66, 67, 77, 78, 81]. Of these, seven studies [50, 55, 58, 59, 65, 66, 78] additionally reported the prevalence of anxiety or its symptoms. No study investigated the prevalence of anxiety or its symptoms as a stand‐alone mental disorder among YLWH. Tables 2 and 3 present the prevalence of depressive and anxiety disorders (or their symptoms according to the screening tool used), as reported from the above studies.

In summary, wide‐ranging prevalence estimates of CMDs were reported among YLWH. The prevalence of major depression ranged between 16.0% and 40.8% [45, 46, 50, 64]. A prevalence of 5.2% for symptoms of major depression was reported by a Ugandan study [55]. When comparing YLWH and their HIV‐negative peers [65], the observed prevalence of symptoms of major depression was 6.2% and 7.4% respectively. Regardless of the screening tool used, depressive symptoms ranged between 4.4% and 52.6%. The prevalence of anxiety disorder among YLWH from Uganda was 45.6% [50]. When comparing YLWH and their HIV‐negative peers [65], the observed prevalence of symptoms of panic disorder was 3.7% and 2.5% respectively. Regardless of the screening tool used, anxiety symptoms ranged between 2.2% and 25.0%. Wide‐ranging CMD prevalence estimates have been documented in past reviews involving YLWH [11, 12, 91] but also adults living with HIV in SSA [9, 38]. Differences in study context and population (like the conceptualization of mental health issues, exposure levels to triggers of mental health problems), study respondents (self vs. others) and use of heterogenous measurement tools (including different cut‐off scores for similar measures) may contribute to the wide variation of the reported prevalence estimates. As a start point for possible quantification of the magnitude of CMDs among YLWH in SSA, researchers, perhaps from similar regions, should work towards the use of homogenous mental health measurement tools.

Compared to HIV‐negative peers, YLWH from SSA appear to be experiencing higher CMDs, particularly depressive disorders. However, the evidence is limited to draw any conclusions. Only five studies in this review [46, 52, 65, 66, 82] compared CMDs between YLWH and their HIV‐negative peers. Four of these studies reported significantly higher prevalence estimates of either major depression [46] or depressive symptoms [52, 66, 82] among YLWH (Table 2). In these studies, YLWH were 3.5‐ [46], 2.9‐ [66], 6.0‐ [82] and 2.1‐times [52] more likely to have higher depression compared to their HIV‐negative peers. Even though the odds of symptoms of major depression were 17% less likely among YLWH compared to HIV‐negative peers in the fifth study [65], there was no significant between‐group difference (Table 2). In the literature, individual empirical studies from other settings comparing for instance depressive symptoms among YLWH and their HIV‐negative peers report mixed results. Some observe significant group differences [92], whereas others observe insignificant differences [27, 93].

For anxiety, two of the five studies above [65, 66] also compared the prevalence of anxiety symptoms between YLWH and their HIV‐negative peers and reported slightly higher, but statistically insignificant, prevalence estimates among YLWH (Table 3). In these studies, even though insignificant, the odds of anxiety symptoms were 1.5 [65] and 1.3 times [66] higher in YLWH. In contrast, a study from Italy found significantly higher anxiety scores among YLWH compared to their HIV‐negative peers [92]. For a clearer insight as to whether YLWH from SSA are at an elevated risk of CMDs compared to their HIV‐negative peers, there is a need for more comparative research.

Despite the observed wide‐ranging prevalence estimates, this review generally shows that the burden of CMDs among YLWH from SSA is high, and that rates may be two to six times higher when compared with HIV‐negative youths, particularly for depressive disorders. However, caution must be taken when interpreting the reported prevalence estimates. Only four studies [45, 46, 50, 64] used a mental health diagnostic interview based on either DSM or ICD criteria. Two studies [55, 65] used DSM‐referenced checklists to assess symptoms of major depression and an anxiety disorder. The rest collected mental health data of YLWH using screening tools, some with unknown reliability and/or validity. Most importantly, screening and early management or referral for treatment of CMDs among YLWH from SSA are urgently needed at the HIV clinics servicing these youths, more so because CMDs co‐occurring with HIV are associated with worse HIV outcomes [23, 25].

Overall, fewer studies in this review focussed on anxiety compared to depression. This under‐investigation of anxiety disorders is of concern because previous research involving YLWH report higher rates of anxiety than depression [94]. Partly, the under‐investigation could be due to the paucity of adequately validated measurement tools of anxiety [95]. To allow more research focus on anxiety among YLWH in the African context, as has depression, there is a need for adequate validation of measurement tools for anxiety, taking into consideration contextual and cultural differences within the SSA setting.

### 3.4 Correlates of CMDs among YLWH from SSA

To the best of our knowledge, this is the first review from SSA to comprehensively collate information about factors associated with CMDs in YLWH. Recent reviews from SSA involving YLWH provide an overview of some of these factors because of broadly covering multiple psychiatric disorders [13, 14], focussing on only one correlate – ART adherence [14] or including HIV‐positive young people up to 19 years only [13, 14]. In this review, 19 studies reported the correlates of either major depression [64], symptoms of major depression [65] or depressive symptoms among YLWH [28, 48, 49, 53, 54, 56, 58, 61, 62, 66, 67, 69, 77, 78, 81, 82, 84]. Of these, four studies concurrently investigated correlates of anxiety symptoms [58, 66, 69, 78]. One study [68] independently focused on the correlates of anxiety symptoms. There was limited consensus across studies for most of the reported correlates. Generally, the factors reported to be significantly associated with CMDs among YLWH from these studies can be categorized into sociodemographic, psychosocial and HIV‐related clinical correlates and are presented as such in this paper.

### 3.5 Correlates of major depression or depressive symptoms among YLWH from SSA

Table 4 presents in detail the identified correlates of major depression or depressive symptoms among YLWH as reported from the studies above. In summary, none of the studies reported any sociodemographic correlate of major depression. The sociodemographic factors that significantly increased the risk for higher depressive symptoms among YLWH included: older age [49, 53, 69, 77, 84], female sex [28, 53], fewer schooling years [28, 49], longer distance to the clinic [54], and HIV‐positive status [66, 82]. Similarly, but in the inverse direction, Haas *et al*. [78] report younger age as significantly lowering the risk for depressive symptoms. There appeared to be a consensus between two or more studies regarding older age, female sex, fewer schooling years and HIV‐positive status as significant sociodemographic correlates of higher depressive symptoms, congruent with results from previous reviews involving YLWH [5, 11, 12]. In contrast, one included study [81] reported older youth age as protective against depressive symptoms and male sex as a risk indicator for higher depressive symptoms. Better overall health [69], residing in rural areas [82], not being in a romantic relationship [28], not failing a term or class [28] and higher height for age z‐scores [67] were the sociodemographic factors that significantly decreased the risk for depressive symptoms. However, there was a lack of consensus across studies for these correlates.

**Table 4:** Sociodemographic, psychosocial and HIV‐related correlates of major depression or depressive symptoms among young people living with HIV from SSA

Author, year	Outcome	Measure of effect (precision)	Sociodemographic correlates		Psychosocial correlates		HIV‐related correlates	
			Risk indicators	Protective indicators	Risk indicators	Protective indicators	Risk indicators	Protective indicators
Abebe *et al*., 2019 [84]	Depressive symptoms	AOR (95% CI)	‐Older age: 2.20 (1.33 to 3.62)	NR	‐Low social support: 2.74 (1.42 to 5.27) ‐HIV‐related stigma: 2.06 (1.35 to 3.14)	NR	‐Poor ART adherence: 1.73 (1.13 to 2.64) ‐History of opportunistic infections: 1.94 (1.15 to 3.27)	NR
Ashaba *et al*., 2018 [64]	Major depression	AOR (95% CI)	NR	NR	‐Bullying: 1.09 (1.00 to 1.20)	NR	NR	NR
Boyes *et al*., 2018 [69]	Depressive symptoms	β (Se)	‐Older age: 0.07 (0.02)	‐Better overall health: −0.18 (0.08)	‐Internalized stigma: 0.29 (0.05) ‐Negative clinic interactions: 0.06 (0.02) ‐Emotional abuse 0.56 (0.14) ‐Sexual abuse: 0.83 (0.25) ‐Bullying victimisation: 0.04 (0.02)	‐Self‐efficacy: −0.04 (0.02) ‐Higher social support: −0.11 (0.020) ‐Access to a clinic support group: −0.32 (0.10) ‐Positive parenting: −0.04 (0.01)	‐ART side effects: 0.49 (0.12)	NR
Buckley *et al*., 2020 [65]	Symptoms of Major Depression	AOR (95% CI)		NR	‐Living with someone with aggression or anger problems: 2.80 (1.05 to 7.44) ‐Ever witnessing violence at home: 4.34 (1.65 to 11.46)	NR	NR	NR
Cavazos‐Rehg *et al*., 2020 [81]	Depressive symptoms	AOR (95% CI)	‐Male sex: 1.62 (1.15 to 2.27)	‐Older age: 0.87 (0.77 to 0.98)	‐Grandparent as primary caregiver: 1.83 (1.16 to 2.88)	‐Higher social support (from friends): 0.96 (0.91 to 0.998) ‐Higher socio‐economic status (additional assets and employment): 0.85 (0.74 to 0.99) ‐Family cohesion: 0.94 (0.91 to 0.96)	NR	NR
Dow *et al*., 2016 [53]	Depressive symptoms	MR (95% CI)	‐Older age: 1.08 (1.03 to 1.14) ‐Female sex: 1.52 (1.11 to 2.09)	NR	‐HIV‐related stigma: 1.08 (1.04 to 1.11)	NR	‐Poor ART adherence: 1.52 (1.07 to 2.18)	NR
Earnshaw *et al*., 2018 [61]	Depressive symptoms	ARR (95% CI)		NR	‐Internalized stigma: 1.23 (1.13 to 1.34) ‐Associative stigma: 1.59 (1.37 to 1.84)	NR	NR	NR
Ekat *et al*., 2020 [49]	Depressive symptoms	APR (95% CI)	‐Older age: 2.07 (1.06 to 4.04) ‐Stopping education: 1.60 (1.06 to 2.42)	NR	NR	NR	‐Poor ART adherence: 2.06 (1.23 to 3.45)	NR
Filiatreau *et al*., 2020 [62]	Depressive symptoms	APR (95% CI)	NR	NR	‐History of physical violence: 2.02 (1.43 to 2.84) ‐History of sexual violence: 2.25 (1.58 to 3.19) ‐History of physical or sexual violence: 2.01 (1.43 to 2.83) ‐History of physical and sexual violence: 3.01 (2.06 to 4.39)	NR	NR	NR
Gaitho *et al*., 2018 [77]	Depressive symptoms	AOR (95% CI)	‐Older age: 2.34 (1.40 to 4.00)	NR	NR	NR	‐Poor ART adherence: 1.84 (1.08 to 3.10)	NR
Haas *et al*., 2020 [78]	Depressive symptoms	AOR (95% CI)	NR	‐Younger age: 10 to 12 years age group vs. 16 to 19 years age group = 0.05 (0.01 to 0.21) 13 to 15 years age group vs. 16 to 19 years group = 0.18 (0.08 to 0.40)	‐Conflict in the household: 3.76 (1.97 to 7.17)	NR	NR	NR
Hoare *et al*., 2019 [66]	Depressive symptoms	β (95% CI)	‐HIV+ status: 5.08 (1.35 to 8.82)	NR	‐Stressful life events: 0.83 (0.57 to 1.08) ‐HIV‐related stigma: 9.93 (2.88 to 16.98)	NR	NR	NR
Kemigisha *et al*., 2019 [54]	Depressive symptoms	AOR (95% CI)	‐Travelling >30 minutes for routine clinic care: 1.66 (1.02 to 2.70)	NR	NR	NR	NR	NR
Kikuchi *et al*., 2017 [67]	Depressive symptoms	AOR (95% CI)	NR	‐Higher height‐for‐age: 0.78 (0.62 to 0.99)	‐Caregiver depression: 1.79 (1.13 to 2.7)	NR	‐Taking efavirenz: 2.33 (1.21 to 4.50)	NR
Kim *et al*., 2015 [28]	Depressive symptoms	β (95% CI)	‐Female sex: 2.13 (0.82 to 3.43) ‐Fewer years of schooling: 3.84 (1.71 to 5.98)	‐Not failing a school term/class: −1.46 (−2.76 to −0.17) ‐Not being in a romantic relationship: −2.38 (−4.35 to −0.41)	‐Being bullied for taking ART: 5.31 (3.19 to 7.43)	‐No death in the family: −1.77 (−3.15 to −0.39) ‐Satisfaction with physical appearance: −0.93 (−1.74 to 0.11)	NR	‐No immune suppression: −2.58 (−4.29 to −0.87)
Lwidiko *et al*., 2018 [82]	Depressive symptoms	AOR (95% CI)	‐HIV+ status: 1.96 (1.1 to 3.45)	‐Rural residence: 0.61 (0.39 to 0.96)	‐History of childhood deprivation: 4.76 (2.79 to 8.13)	NR	NR	NR
Okawa *et al*., 2018 [56]	Depressive symptoms	AOR (95% CI)	NR	NR	‐Unsatisfactory relationship with family: 3.01 (1.20 to 7.56) ‐Unsatisfactory relationship with health workers: 2.68 (1.04 to 6.93) ‐HIV‐related stigma: 2.99 (1.07 to 8.41)	NR	NR	NR
West *et al*., 2018 [58]	Depressive symptoms	APR (95% CI)	NR	NR	NR	‐Higher social support: 0.25 (0.10 to 0.59)	NR	NR
Yarhere & Jaja, 2020 [48]	Depressive symptoms	β (t)	NR	NR	‐Insomnia: 5.61 (2.94) ‐Suicidal thoughts: 4.64 (3.39)	NR	NR	Higher CD4 count: −0.001 (2.74)

AOR, Adjusted odds ratio; APR, Adjusted prevalence ratio; ART, Antiretroviral therapy; CD4, Cluster of Differentiation‐4; CI, Confidence interval; MR, Mean ratio; NR, None reported; Se, Standard error; t, t statistic; β, Beta coefficients (adjusted).

Females living with HIV from SSA could be at a higher risk of depressive symptoms because of additional experiences of traumatic events such as sexual abuse and intimate partner violence, some of which may have had a role in their acquisition of HIV infection [96, 97]. Additionally, they are more likely to be stigmatized [98] and blamed for HIV transmission within families in patriarchal societies like those of SSA [99]. Older YLWH compared to younger ones are more likely to understand the threat posed by HIV infection to their own life [16], which may manifest as depressive symptoms. The awareness of HIV‐related cognitive deficits [41] by a YLWH manifesting in ways like grade retention or poor performance may explain the association between fewer years of schooling and higher depressive symptoms. Although two studies in this review observed significant associations between HIV‐positive status and higher depressive symptoms, Western empirical studies report non‐significant associations [27, 93]. In SSA where poverty is high [100], HIV‐related adjustments such as recommended intake of a balanced diet with ART use and meeting the regular transportation costs for clinic appointments may be additional challenges to most families of YLWH. Such challenges may lead to psychiatric manifestations among YLWH.

Bullying was the only reported psychosocial correlate of major depression in the study examining the relationship between psychosocial factors and major depression [64]. Psychosocial factors that significantly increased the risk for higher symptoms of major depression among YLWH included living with someone who has anger/aggression problems and ever witnessing violence at home [65]. Psychosocial factors that significantly increased the risk for higher depressive symptoms among YLWH were as follows: bullying victimization [69], bullying for taking ART [28], caregiver depression [67], grandparent as primary caregiver [81], low social support [84], HIV‐related stigma in its various forms such as perceived stigma [53, 56, 66, 84], internalized stigma [61, 69] and associative stigma [61], history of sexual [62, 69], emotional [69] and physical abuse [62] or a combination of physical and/or sexual abuse [62], conflict in the household [78], unsatisfactory relationship with family or health workers [56], insomnia and suicidal ideation [48], negative clinic interactions [69], history of childhood deprivation [82] and stressful life events [66]. On the other hand, the following psychosocial factors significantly lowered the risk for depressive symptoms in YLWH: access to a clinic support group [69], positive parenting [69], higher socio‐economic status [81], family cohesion [81], not being bereaved in the family [28], self‐efficacy [69], satisfaction with physical appearance [28] and higher social support [58, 69, 81]. Among the reported psychosocial correlates of depressive disorders in YLWH, bullying, HIV‐related stigma, history of sexual abuse and social support were consistently reported across two or more studies as significantly associated with depressive disorders, similar to previous review findings [5, 11, 12].

The negative effects of HIV‐related stigma among YLWH including social devaluation and experience of injustices like restrictions in interacting with other people and being denied equal opportunities of enrolling or staying in school [20], may explain why HIV‐related stigma (in its various forms) is associated with higher depressive symptoms in YLWH. HIV‐related stigma can lead to depression manifesting as low self‐worth, self‐isolation, loss of hope in future plans or aspirations and poor ART adherence [20, 84, 101]. As previously emphasized [19, 20], there is a need for continuously addressing HIV‐related stigma in multiple settings within the community. Bullying can lead to negative outcomes such as humiliation, self‐blame and shame [102] and coupled with living with HIV at a younger age, high levels of depressive symptomatology may be expected among YLWH. The finding that higher social support is associated with fewer depressive symptoms (or vice versa, low social support being associated with more depressive symptoms) supports the proposition of a buffering effect of social support against psychological distress as described in the “buffering hypothesis” [103]. This hypothesis postulates that any form of social support (social companionship, emotional, informational, or instrumental support) proffers protection to individuals facing stressful life events (in this case, HIV infection adversity) and assists them in coping with distress. The persistent psychological distress among sexually abused YLWH can be addressed through continued counselling and appropriate support mechanisms.

The following HIV‐related clinical factors significantly increased the risk for elevated depressive symptoms among YLWH: poor adherence to ART [49, 53, 77, 84], history of opportunistic infections [84], experiencing ART side effects [69] and taking efavirenz‐based ART [67]. On the other hand, better immunological stage [28] or increasing CD4 cell count [48] were associated with fewer depressive symptoms among YLWH. See Table 4 for effect sizes reported from individual studies. Of these correlates, only poor adherence to ART was consistently reported across four studies, a finding that has also been observed in past global reviews involving YLWH [5, 11, 12]. Non‐optimal adherence to ART can lead to viral non‐suppression [104], and patients may experience psychological distress when informed of a poor prognosis. This may explain the observed consistent significant association between poor ART adherence and elevated depressive symptoms among YLWH from SSA [49, 53, 77, 84]. However, depression can also be an antecedent to poor ART adherence [25], thus there is uncertainty on the temporality of the significant associations reported in the above four cross‐sectional studies.

### 3.6 Correlates of anxiety symptoms among YLWH from SSA

Table 5 presents in detail the identified correlates of anxiety symptoms among YLWH. The study that diagnosed anxiety disorder [50] did not report any correlation. Younger age [78] and better overall health among YLWH [69] were the only significant sociodemographic correlates of anxiety symptoms. The following psychosocial factors significantly increased the risk for anxiety symptoms among YLWH: internalized stigma [69], anticipated stigma [69], history of sexual abuse or emotional abuse in the past year [69], bullying victimization [69], poor parental monitoring [69], history of physical violence [78] and stressful life events [66]. Conversely, higher social support [58, 68], self‐efficacy [69], positive parenting [69] and access to a clinic support group [69] were the factors that significantly decreased the risk for anxiety symptoms among YLWH from SSA. Experiencing ART side effects was the only significant HIV‐related clinical correlate of anxiety symptoms among YLWH reported by one South African study [69].

**Table 5:** Sociodemographic, psychosocial and HIV‐related correlates of anxiety symptoms among young people living with HIV from SSA

Author (year)	Outcome	Measure of effect (precision)	Sociodemographic correlates		Psychosocial correlates		HIV‐related correlates	
			Risk indicators	Protective indicators	Risk indicators	Protective indicators	Risk indicators	Protective indicators
Besthorn *et al*. (2018) [68]	Anxiety symptoms	β (Se)	NR	NR	NR	‐Higher social support: −0.16 (0.06)	NR	NR
Boyes *et al*. (2018) [69]	Anxiety symptoms	β (Se)	NR	‐Better overall health: −0.18 (0.08)	‐Internalized stigma: 0.54 (0.07) ‐Past year emotional abuse: 1.17 (0.19) ‐History of sexual abuse: 1.08 (0.34) ‐Bullying victimisation: 0.15 (0.02) ‐Poor parental monitoring: 0.02 (0.01) ‐Anticipated stigma: 0.30 (0.08)	‐Self‐efficacy: −0.06 (0.02) ‐Positive parenting: −0.05 (0.01) ‐Access to clinic support group: −0.43 (0.14)	‐ART side effects: 0.51 (0.17)	NR
Haas *et al*., 2020 [78]	Anxiety symptoms	AOR (95% CI)	NR	‐Younger age: 10 to 12 years age group vs. 16 to 19 years age group = 0.05 (0.01 to 0.37) 13 to 15 years age group vs. 16 to 19 years group = 0.19 (0.06 to 0.56)	‐History of physical violence: 2.74 (1.09 to 6.85)	NR	NR	NR
Hoare *et al*. (2019) [66]	Anxiety symptoms	β (95% CI)	NR	NR	‐Stressful life events: 0.72 (0.44 to 1.01)	NR	NR	NR
West *et al*. (2018) [58]	Anxiety symptoms	APR (95% CI)	NR	NR	NR	‐Higher social support: 0.30 (0.13 to 0.71)	NR	NR

APR, Adjusted prevalence ratio; ART, Antiretroviral therapy; CI, Confidence interval; NR, Not reported; Se, Standard error; β, Beta coefficients (adjusted).

Similar to what was observed for depressive symptoms, social support was also a significant correlate of anxiety symptoms reported across two studies [58, 68]. As earlier noted, social support may also provide a buffering effect against anxiety symptoms [103].

### 3.7 Quality of included studies

(Additional file S3) shows the quality scores of included studies according to their study designs, based on the Newcastle‐Ottawa risk of bias assessment tool. Two studies were graded to be of very good quality [55, 69], twelve of good quality [28, 78, 82, 84], nine of satisfactory quality [45, 49, 50, 51, 56, 61, 62, 68, 81] and seven of unsatisfactory quality [46, 47, 48, 57, 59, 60, 63]. One study [52], available only as an abstract publication, provided relevant mental health data for this review. This study was rated as unclear of high risk of bias because of a lack of feedback from authors when contacted (on three attempts) with a request for data that would enable assessment of the quality of the entire study.

Even though most of the studies reporting on correlates of CMDs among YLWH were of low risk of bias, they were cross‐sectional in design. This study design limits inferences on causality. Therefore, the summarized significant correlates of CMDs among YLWH in this review should be interpreted with caution. For better decisions on priority intervention areas, future studies from this setting should seek to substantiate the causal direction of the identified correlates, using, for instance longitudinal study designs.

### 3.8 Study limitations

This review has several limitations worth highlighting. First, the review does not provide pooled prevalence estimates of CMDs among YLWH from SSA because measurement tools used across studies were highly heterogeneous. Relatedly, because a meta‐analysis could not be performed, we are unable to report on publication bias. Second, the review deliberately focused on SSA. Even though the region has predominantly low‐ to middle‐income countries, findings may not necessarily be generalizable to other low‐ and middle‐income countries outside this context. Relatedly, across the different countries in SSA, there is diversity in aspects such as language, religious and cultural practices which may make the results ungeneralizable to some communities within the region. Lastly, the review search strategy was biased as only publications in English were considered. It may be that we left out important work reported in a language other than English.

### 3.9 Implications of the findings for future research, policy and practice

The limitations notwithstanding, this study has important implications for future research, policy and practice. There is a need to invest in mental health awareness as one of the primary prevention strategies aiming at preventing the occurrence of CMDs at high rates among YLWH in SSA. This can entail psychoeducating YLWH about CMDs, that is what they are, the signs and symptoms, when and where to seek help, and providing them with self‐help tips or quick guides through forums such as peer‐to‐peer meetings. The high burden of CMDs in YLWH from SSA highlights the urgent need to test youth‐friendly psychological and psychosocial interventions that address CMDs faced by African youths living with HIV. Adaptation of available interventions such as those identified in a scoping review by Okonji *et al*. [105] may be a good starting point. The high burden also calls for the integration of mental healthcare into the existing HIV care packages offered to YLWH in this setting. Successful integration requires training of primary health care personnel at the HIV clinics on how to manage CMDs (using, e.g. the World Health Organization’s mhGAP intervention guide [106]), adequate infrastructure (including the availability of psychotropic medication), followed by regular supervision and support from mental health specialists using a task‐shifting approach [107, 108].

Many of the included studies were cross‐sectional in design, did not compare the burden of CMDs between YLWH and their uninfected peers, and focused more on depression than anxiety. Alternative study designs that ascertain causal relationships are recommended for future investigations of factors associated with CMDs among YLWH in SSA. Where feasible, future studies seeking to understand CMDs in YLWH should include an appropriate comparison group of HIV‐negative peers to clearly describe the burden. Finally, more research on anxiety among YLWH from SSA is needed. Currently, this data remains limited.

## 4 Conclusions

According to this review, the prevalence of CMDs in YLWH from SSA is substantially high despite the wide variation of reported estimates. From studies that recruited a comparison group of HIV‐negative peers, it appears YLWH are at a higher risk of experiencing CMDs particularly depressive symptoms, but more comparative research is needed to draw definite conclusions. The mental health experience of YLWH in SSA is not any different compared to that of YLWH from other settings, all are reporting high rates of CMDs. However, some of the factors associated with CMDs among YLWH in SSA are context‐specific and may require contextualized intervention approaches. YLWH at an elevated risk of CMDs in SSA such as females, older youths, those with fewer schooling years, with a history of sexual abuse, reporting ART adherence issues, being bullied or experiencing HIV‐related stigma may benefit from early management or referral for treatment using a stepped care approach [109] if at least targeted screening for CMDs is done at the youth HIV clinics. Social support may lower the risk for CMDs among YLWH in SSA and can be an important component to consider when designing youth‐friendly intervention packages for YLWH with comorbid CMDs.

## Competing interests

The authors declare no conflict of interest.

## Authors’ contributions

MKN, AA and CRJCN conceived the study. ET, MKN, AA, CRJCN designed the study. CN, HK, PC contributed to the design of the study. ET did the initial screening of the articles, whereas MKN and CN independently checked the quality of data screening. ET and MKN independently extracted data and assessed the risk of bias for the included studies. ET and MKN wrote the first draft of the manuscript. AA, CN, CRJCN, HK and PC critically reviewed subsequent versions of the manuscript. All the authors have approved the submission of this final version.

## Abbreviations

ART, antiretroviral therapy; CASI‐5, child and adolescent symptom inventory – fifth edition; CD4, cluster of differentiation‐4; CMDs, common mental disorders; DSM, diagnostic and statistical manual of mental disorders; ICD‐10, international classification of diseases – tenth edition; MINI‐KID, mini internatonal neuropsychiatric interview for children and adolescents; PRISMA, preferred reporting items for systematic reviews and meta‐analysis; PROSPERO, the international prospective register of systematic reviews; SSA, sub‐Saharan Africa; YI‐4R, Youth inventory – fourth revision; YLWH, young people living with HIV.

## Funding

This work was funded by the Wellcome Trust International Master’s Fellowship to MKN (Grant number 201310/Z/16/Z). During this project, ET was supported by DELTAS Africa Initiative [DEL‐15‐003]. The DELTAS Africa Initiative is an independent funding scheme of the African Academy of Sciences (AAS)'s Alliance for Accelerating Excellence in Science in Africa (AESA) and supported by the New Partnership for Africa's Development Planning and Coordinating Agency (NEPAD Agency) with funding from the Wellcome Trust (107769/Z/10/Z) and the UK government. AA holds an award (Grant number MR/M025454/1) jointly funded by the UK Medical Research Council (MRC) and the UK Department for International Development (DFID) under MRC/DFID concordant agreement and is also part of the EDCTP2 programme supported by the European Union. The funders did not have a role in the design and conduct of the study. The views expressed in this publication are those of the author(s) and not necessarily those of AAS, NEPAD Agency, Wellcome Trust or the UK government.

## Supporting information


**Additional file S1**. Search string used in PubMed database.

**Additional file S2**. Characteristics of included studies.

**Additional file S3**. Quality scores of the included studies.
