# Supplementary material for: Prevalence and factors associated with common mental disorders in young people living with HIV in sub‐Saharan Africa: a systematic review
Source: J Int AIDS Soc. 2021 Jun 24;24(Suppl 2):e25705. doi: 10.1002/jia2.25705 (PMC8222842; doi:10.1002/jia2.25705)
Supplement: Supplementary file 2 — Additional file S2. Characteristics of included studies. [file JIA2-24-e25705-s001.docx]

*Table 1: Summary of included studies reporting both the prevalence and correlates of common mental disorders among young people living with HIV from sub-Saharan Africa.*

| **Author (year)** | **Country** | **Demographic data** | | **Clinical data** | | **Study characteristics** | | | | | |
| --- | --- | --- | --- | --- | --- | --- | --- | --- | --- | --- | --- |
|  |  | **Age range (years)** | **Female (%)** | **Treatment status** | **ART regimen** | **Study design** | **Sample source** | **Study setting** | **Sampling method** | **Sample size (N)** | **Study limitations as noted by authors** |
| Abebe et al., 2019 [[84](#_ENREF_84)] | Ethiopia | 15-24 | 53.6 | On ART | -1^st^ line (80.7%)  -2^nd^ line (19.3%) | Cross-sectional | -Nine hospitals with HIV care | Urban | Systematic random | 507 | - Cross-sectional study design limits causal inferences.  - Social desirability bias from self-report measures. |
| Ashaba et al., 2018 [[64](#_ENREF_64)] | Uganda | 13-17 | 58 | On ART | NR | Cross-sectional | -HIV clinic within a referral hospital | Rural | Consecutive sampling | 224 | - Cross-sectional study design precludes causal inferences.  -Non-generalizability to other youth groups outside 13-17 years  -Sampling bias. |
| Buckley et al., 2020 [[65](#_ENREF_65)] | South Africa | 15-18 | 61 | On ART | NR | Cross-sectional | -Paediatric wellness HIV clinics for the HIV+  -HIV research unit and the community for the HIV- | Urban | NR | 162 Total  81 HIV+  81 HIV- | -Cross-sectional design precludes causal inferences.  -Small sample size may have precluded useful analysis.  -Probable social desirability bias.  -Some factors that may have explained the findings were not known. |
| Cavazos-Rehg et al., 2020 [[81](#_ENREF_81)] | Uganda | 10-16 | 56.4 | On ART | NR | Cross-sectional | -HIV clinics | NR | NR | 675 Total  592 In-school  83 Out-of-school | -Probable social desirability bias.  -Cross-sectional design precludes causal inferences.  -Adolescents may not have been fully aware of social and economic conditions impacting their families.  -Limited statistical power to analyse data on out-of-school adolescents. |
| Dow et al., 2016 [[53](#_ENREF_53)] | Tanzania | 12-24 | 54.4 | Mixed | -1^st^ line (57.7%)  -2^nd^ line (34.1%)  -ART naïve (8.2%) | Cross-sectional | -HIV youth-focused clinic | NR | NR | 182 | -Cross-sectional study design precludes causal inferences.  -Tools not locally validated.  -Probable interviewer reporting and social desirability bias. |
| Earnshaw et al., 2018 [[61](#_ENREF_61)] | South Africa | 13-24 | 54.4 | NR | NR | Cross-sectional | -HIV research unit within a hospital | Urban | NR | 250 | -Cross-sectional study design precludes causal inferences.  -Convenience sampling may limit generalizability to other YLWH. |
| Ekat et al., 2020 [[49](#_ENREF_49)] | DRC | 10-19 | 50 | On ART | -EFV-based (38%)  -NVP-based (50%)  -Other (12%) | Cross-sectional | -HIV care clinics | Urban | NR | 135 | -Cross-sectional design precludes causal inferences.  -Inability to use viral load as a measure of adherence. |
| Filiatreau et al., 2020 [[62](#_ENREF_62)] | South Africa | 12-24 | 71.3 | NR | NR | Cross-sectional | -HIV clinics | Rural | NR | 334 | -Potential underestimation of prevalence estimates.  -Cross-sectional design may have introduced recall bias.  -Non-generalizability of results. |
| Gaitho et al., 2018 [[77](#_ENREF_77)] | Kenya | 10-19 | 46.3 | On ART | NR | Cross-sectional | -HIV clinic within a referral hospital | Urban | NR | 270 | -Cross-sectional study design precludes causal inferences.  -Possible social desirability bias.  -Lack of a comparison group.  -Tools not locally validated. |
| Haas et al., 2020 [[78](#_ENREF_78)] | South Africa | 10-19 | 49.5 | On ART | -NNRTI-based (64.2%).  -PI-based (34.4%)  -Other (1.4%) | Cross-sectional | -HIV clinic within a public hospital | Urban | NR | 1088 | -Use of non-validated screening tools.  -Possible underreporting and social desirability bias. |
| Hoare et al., 2019 [[66](#_ENREF_66)] | South Africa | 9-11 | 51.6 | On ART | -1^st^ line (80%)  -2^nd^ line (15.7%)  -3^rd^ line (4.3%) | Cross-sectional | -HIV Research centre within a hospital | Urban | NR | 248 Total  204 HIV+  44 HIV- | -Cross-sectional study design precludes causal relationships. |
| Kemigisha et al., 2019[[54](#_ENREF_54)] | Uganda | 10-19 | 62.2 | Mixed  -On ART (95.2%)  -ART naïve (4.8%) | -NVP-based (48.5%).  -EFV-based (32.4%). | Cross-sectional | -HIV clinics within a referral hospital and a health centre | Rural and peri-urban | NR | 336 | -Possible selection bias.  -Cross-sectional study design precludes causal inferences.  - Actual duration of depression could not be ascertained as the tool only screened for depressive symptoms in the past 2 weeks.  -Tool used not well validated in young adolescents. |
| Kikuchi et al., 2017 [[67](#_ENREF_67)] | Rwanda | 7-14 | 49.1 | On ART | -EFV-based (23.8%) | Cross-sectional | -HIV clinic within a health facility | Urban | Convenience sampling | 475 | -Convenience sampling.  -Lack of control for other potential confounding factors. |
| Kim et al., 2015 [[28](#_ENREF_28)] | Malawi | 12-18 | 56 | On ART | NR | Cross-sectional | -HIV clinics within 2 referral hospitals | Urban | Convenience sampling | 562 | -Findings may not be generalizable to all adolescents.  -Lack of control group limits drawing of conclusions about prevalence estimates. |
| Lwidiko et al., 2018 [[82](#_ENREF_82)] | Tanzania | 7-17 | 58 | On ART | NR | Case-control | -HIV clinic | Urban | Systematic and multistage sampling | 900 Total  300 HIV+  600 HIV- | -Non-generalizability of results to other regions as participants were from single district.  -Other factors likely to be associated with depressive symptoms not considered.  -HIV status of control group not verified. |
| Okawa et al., 2018 [[56](#_ENREF_56)] | Zambia | 15-19 | 57.9 | Mixed  -On ART (94.2%)  -ART naïve (5.8%) | NR | Cross-sectional | -HIV clinics within a teaching hospital | Urban | Convenience sampling | 190 | -Possible under-estimation of findings.  -Limited validity and reliability of findings.  -Focus was limited to participants in late adolescence.  -Non-generalizability of findings to other settings. |
| West et al., 2018 [[58](#_ENREF_58)] | South Africa | 9-19 | 53.2 | Mixed  -On ART (88.4%)  -ART naïve (11.6%) | NR | Cross-sectional | -HIV clinic | Urban | NR | 278 | -Cross-sectional study design precludes causal inferences.  -Lack of comparison group.  -Social desirability bias. |
| Yarhere & Jaja, 2020 [[48](#_ENREF_48)] | Nigeria | 10-18 | 12.1 | NR | NR | Cross-sectional | -HIV clinic within a teaching hospital | NR | NR | 58 | NR |
| **Note:** ART- Antiretroviral therapy  EFV- Efavirenz  DRC- Democratic Republic of Congo  NR- Not Reported  NVP- Nevirapine  PI- Protease inhibitors  YLWH- Young People Living with HIV | | | | | | | | | | | |

Table 2: Summary of included studies reporting only the prevalence of common mental disorders among young people living with HIV from sub-Saharan Africa**.**

| **Author (year)** | **Country** | **Demographic data** | | **Clinical data** | | **Study characteristics** | | | | |  |
| --- | --- | --- | --- | --- | --- | --- | --- | --- | --- | --- | --- |
|  |  | **Age range (years)** | **Female (%)** | **Treatment status** | **ART regimen** | **Study design** | **Sample source** | **Study setting** | **Sampling method** | **Sample size (N)** | **Study limitations as noted by the authors** |
| Adeyemo et al., 2020 [[45](#_ENREF_45)] | Nigeria | 10-19 | 38.2 | NR | NR | Cross-sectional | HIV clinics within a hospital and a research unit | Urban | Convenience sampling | 201 | -Cross-sectional design precludes causal inferences.  -Possible social desirability bias. |
| Bankole et al., 2017 [[46](#_ENREF_46)] | Nigeria | 8-16 | 58.7 | Mixed  -On ART (89.3%) | NR | Cross-sectional | HIV clinic within a tertiary care facility | Urban | Consecutive sampling | 150 Total  75 HIV+  75 HIV- | -Cross-sectional study design precludes causal inferences.  -Non-generalizability of study findings to other young people. |
| Dyer et al., 2020 [[60](#_ENREF_60)] | Kenya | 10-24 | 65 | NR | NR | Cross-sectional | -HIV clinics within 9 health facilities | NR | NR | 479 | NR |
| Fawzi et al., 2016 [[51](#_ENREF_51)] | Rwanda | 10-17 | 50 | On ART | -Adult 1^st^ line for youths >15 years  - 1^st^ line child therapy for all children ≤15 years | Cross-sectional | Community | Rural | Stratified random | 193 | -Small sample size hence wider confidence intervals.  -Potential underestimation of adherence rates from caregiver and self-reports.  -Limited generalizability to other age groups and settings. |
| Kinyanda et al., 2019 [[55](#_ENREF_55)] | Uganda | 12-17 | 52.8 | Mixed  -On ART (94.6%)  -ART naïve (5.4%) | NR | Cross-sectional | 5 youth HIV clinics | Urban  & Rural | NR | 479 | -Cross-sectional study design precludes causality.  -Sampling bias.  -Low validity and reliability of screening instruments. |
| Molinaro et al., 2019 [[52](#_ENREF_52)] | Zambia | 8-17 | 50 | On ART | NA | Cohort | NA | NA | NA | 400 Total  200 HIV+  200 HIV- | NA |
| Musisi & Kinyanda, 2009 [[50](#_ENREF_50)] | Uganda | 8-18 | 55.6 | On ART | NR | Cross-sectional | HIV healthcare centre | Urban | Consecutive sampling | 82 | -Non-generalizability to the wider youth population.  -Exclusion of those not informed of their HIV status. |
| Ramos et al., 2018 [[57](#_ENREF_57)] | Tanzania | 11-24 | 55.4 | Mixed  -On ART (92.8%)  -ART naïve (7.2%) | -NNRTI-based (68.2%)  -PI-based (24.6%)  -ART naïve (7.2%) | Cross-sectional | HIV clinic within a health centre and a referral hospital | Urban | NA | 280 | -Tool not locally validated.  -Non-generalizability to YLWH who declined participation. |
| Paul et al., 2015 [[63](#_ENREF_63)] | Zambia | 11-17 | 66 | NR | NR | Cross-sectional | HIV clinic within a referral hospital | Urban | Systematic random | 100 | NR |
| Sale & Gadanya, 2008 [[47](#_ENREF_47)] | Nigeria | 15-25 | 47.5 | On ART | NR | Cross-sectional | HIV clinic and medical wards within a hospital | Urban | NR | 162 | -Uncertainty about data quality as some respondents may have had psychotic features making their responses unequivocal or necessitated input from their relatives. |
| Woollett et al., 2017 [[59](#_ENREF_59)] | South Africa | 13-19 | 52 | Mixed  -On ART (88%).  -ART naïve (12%) | NR | Cross-sectional | 5 paediatric HIV clinics within a hospital, health centre, and primary healthcare clinic | Urban | Convenience sampling | 343 | -Convenience sampling.  -Reporting and social desirability bias.  -Lack of contextual standardization of scores and instruments.  -No comparison groups.  -No precise test to determine perinatal infection.  -Not generalizable to adolescents in the general population. |
| **Note:** ART- Antiretroviral therapy  NA- Not Available. This study was only available as an abstract submission to the annual American Academy of Neurology (AAN) meeting. Extracted information relevant to this  the review was based on only the abstract  NNRTI- Non-nucleoside reverse transcriptase inhibitors  NR- Not Reported  PI- Protease inhibitors | | | | | | | | | | | |

*Table 3: Summary of included studies reporting correlates of common mental disorders among young people living with HIV from sub-Saharan Africa.*

| **Author (year)** | **Country** | **Demographic data** | | **Clinical data** | | **Study characteristics** | | | | |  |
| --- | --- | --- | --- | --- | --- | --- | --- | --- | --- | --- | --- |
|  |  | **Age range (years)** | **Female (%)** | **Treatment status** | **ART regimen** | **Study design** | **Source of sample** | **Study setting** | **Sampling method** | **Sample size (N)** | **Study limitations as noted by the authors** |
| Besthorn et al., 2018 [[68](#_ENREF_68)] | Namibia | 11-18 | 50 | On ART | NR | Cross-sectional | Paediatric HIV clinic within a hospital | Rural | NR | 132 | -Not generalizable to YLWH from other regions.  -Tool not locally validated.  -Cross-sectional study design limits causal inferences. |
| Boyes et al., 2018 [[69](#_ENREF_69)] | South Africa | 10-19 | 55 | On ART | NR | Cross-sectional | HIV clinic within health facilities | Urban | NR | 1060 | -Cross-sectional study design precludes causal inferences.  -Findings may not be extrapolated to other LMICs as the study was conducted in one country. |
| **Note:** ART- Antiretroviral therapy  LMICs- Low- and middle-income countries  NR- Not Reported | | | | | | | | | | | |
