# Supplementary material for: Prevalence and factors associated with common mental disorders in young people living with HIV in sub‐Saharan Africa: a systematic review
Source: J Int AIDS Soc. 2021 Jun 24;24(Suppl 2):e25705. doi: 10.1002/jia2.25705 (PMC8222842; doi:10.1002/jia2.25705)
Supplement: Supplementary file 3 — Additional file S3. Quality scores of the included studies. [file JIA2-24-e25705-s003.docx]

Table 1: Quality scores of the included cross-sectional studies based on the Newcastle-Ottawa quality assessment tool.

| **First author (publication year)** | **Selection of participants** | | | | **Comparability** | **Ascertainment of Outcome** | | **Total score** | **Quality grade** |
| --- | --- | --- | --- | --- | --- | --- | --- | --- | --- |
|  | **Sample representativeness** | **Sample size** | **Non-respondents** | **Exposure ascertainment** | **Comparability of subjects in outcome groups** | **Outcome assessment** | **Statistical test appropriateness** |  |  |
| Abebe et al., 2019 [[84](#_ENREF_84)] | * | - | * | * | ** | * | * | 7 | Good |
| Adeyemo et al., 2020 [[45](#_ENREF_45)] | - | * | - | * | - | ** | * | 5 | Satisfactory |
| Ashaba et al., 2018 [[64](#_ENREF_64)] | * | * | - | * | ** | ** | * | 8 | Good |
| Bankole et al., 2017 [[46](#_ENREF_46)] | * | - | - | * | - | ** | - | 4 | Unsatisfactory |
| Besthorn et al., 2018 [[68](#_ENREF_68)] | - | - | - | * | ** | ** | * | 6 | Satisfactory |
| Boyes et al., 2018 [[69](#_ENREF_69)] | * | * | * | * | ** | ** | * | 9 | Very good |
| Buckley et al., 2020 [[65](#_ENREF_65)] | - | * | * | * | ** | * | * | 7 | Good |
| Cavazos‑Rehg et al., 2020 [[81](#_ENREF_81)] | - | - | * | - | ** | ** | * | 6 | Satisfactory |
| Dow et al., 2016 [[53](#_ENREF_53)] | - | - | * | * | ** | ** | * | 7 | Good |
| Dyer et al., 2020 [[60](#_ENREF_60)] | - | - | * | - | - | * | * | 4 | Unsatisfactory |
| Earnshaw et al., 2018 [[61](#_ENREF_61)] | - | - | - | * | ** | ** | * | 6 | Satisfactory |
| Ekat et al., 2020 [[49](#_ENREF_49)] | - | - | - | * | ** | * | * | 5 | Satisfactory |
| Fawzi et al., 2016 [[51](#_ENREF_51)] | - | - | - | * | ** | ** | * | 6 | Satisfactory |
| Filiatreau et al., 2020 [[62](#_ENREF_62)] | - | - | * | - | ** | ** | * | 6 | Satisfactory |
| Gaitho et al., 2018 [[77](#_ENREF_77)] | - | * | * | * | ** | * | * | 7 | Good |
| Haas et al., 2020 [[78](#_ENREF_78)] | * | * | * | * | ** | * | * | 8 | Good |
| Hoare et al., 2019 [[66](#_ENREF_66)] | - | * | - | * | ** | ** | * | 7 | Good |
| Kemigisha et al., 2019 [[54](#_ENREF_54)] | - | - | * | * | ** | ** | * | 7 | Good |
| Kikuchi et al., 2017 [[67](#_ENREF_67)] | * | - | - | * | ** | ** | * | 7 | Good |
| Kim et al., 2015 [[28](#_ENREF_28)] | - | - | * | * | ** | ** | * | 7 | Good |
| Kinyanda et al., 2019 [[55](#_ENREF_55)] | * | * | * | * | ** | ** | * | 9 | Very good |
| Musisi & Kinyanda, 2009 [[50](#_ENREF_50)] | * | * | * | * | - | ** | - | 6 | Satisfactory |
| Okawa et al., 2018 [[56](#_ENREF_56)] | - | - | - | * | ** | ** | * | 6 | Satisfactory |
| Ramos et al., 2018 [[57](#_ENREF_57)] | - | - | - | * | - | * | * | 3 | Unsatisfactory |
| Paul et al., 2015 [[63](#_ENREF_63)] | * | - | - | * | - | * | - | 3 | Unsatisfactory |
| Sale & Gadanya, 2008 [[47](#_ENREF_47)] | - | - | - | * | - | ** | - | 3 | Unsatisfactory |
| West et al., 2018 [[58](#_ENREF_58)] | - | - | * | * | ** | ** | * | 7 | Good |
| Woollett et al., 2017 [[59](#_ENREF_59)] | - | - | - | * | - | ** | * | 4 | Unsatisfactory |
| Yarhere & Jaja, 2020 [[48](#_ENREF_48)] | - | - | - | - | ** | * | * | 4 | Unsatisfactory |

Table 2: Quality scores of the included case-control study based on the Newcastle-Ottawa quality assessment tool.

| **First author (publication year)** | **Selection of participants** | | | | **Comparability** | **Ascertainment of exposure** | | | **Total score** | **Quality grade** |
| --- | --- | --- | --- | --- | --- | --- | --- | --- | --- | --- |
|  | **Case definition** | **Representation** | **Control selection** | **Control definition** | **Comparability** | **Exposure ascertainment** | **Same method of ascertainment** | **Non-response rate** |  |  |
| Lwidiko et al., 2018 [[82](#_ENREF_82)] | * | * | * | * | ** | - | * | - | 7 | Good |

Table 3: Quality scores of the included cohort study based on the Newcastle-Ottawa quality assessment tool.

| **First author (publication year)** | **Selection of participants** | | | | **Comparability** | **Outcome** | | | **Total score** | **Quality grade** |
| --- | --- | --- | --- | --- | --- | --- | --- | --- | --- | --- |
|  | **Representativeness of exposed cohort** | **Selection of non-exposed cohort** | **Ascertainment of exposure** | **Demonstration that outcome of interest was not present at start of study** | **Comparability of cohorts on the basis of design or analysis** | **Assessment of outcome** | **Length of follow-up** | **Adequacy of follow-up of cohorts** |  |  |
| Molinaro et al., 2019 [[52](#_ENREF_52)] | Unclear of high risk of bias | | | | | | | | | |
